# Effects of Lifestyle Intervention on Plasma Trimethylamine N-Oxide in Obese Adults

**DOI:** 10.3390/nu11010179

**Published:** 2019-01-16

**Authors:** Melissa L. Erickson, Steven K. Malin, Zeneng Wang, J. Mark Brown, Stanley L. Hazen, John P. Kirwan

**Affiliations:** 1 Integrative Physiology and Molecular Medicine Laboratory, Pennington Biomedical Research Center, Louisiana State University, Baton Rouge, LA 70808, USA; Melissa.Erickson@pbrc.edu; 2 Department of Kinesiology, University of Virginia, Charlottesville, VA 22903, USA; skm6n@virginia.edu; 3 Department of Cellular and Molecular Medicine, Lerner Research Institute, Cleveland Clinic, Cleveland, OH 44106, USA; wangz2@ccf.org (Z.W.); brownm5@ccf.org (J.M.B.); hazen2@ccf.org (S.L.H.)

**Keywords:** trimethylamine N-oxide, obesity, caloric restriction, cardiovascular disease risk factors, gut microbiome, exercise, lifestyle intervention

## Abstract

Accumulating evidence linking trimethylamine N-oxide (TMAO) to cardiovascular disease (CVD) risk has prompted interest in developing therapeutic strategies to reduce its production. We compared two lifestyle intervention approaches: hypocaloric versus eucaloric diet, combined with exercise, on TMAO levels in relation to CVD risk factors. Sixteen obese adults (66.1 ± 4.4 years, BMI (body mass index): 35.9 ± 5.3 kg/m^2^, fasting glucose: 106 ± 16 mg/dL, 2-h PPG (postprandial glucose): 168 ± 37 mg/dL) were randomly assigned to 12 weeks of exercise (5 days/week, 80–85% HR_max_ (maximal heart rate)) plus either a hypocaloric (HYPO) (−500 kcal) or a eucaloric (EU) diet. Outcomes included plasma TMAO, glucose metabolism (oral glucose tolerance test (OGTT) and euglycemic-hyperinsulinemic clamps for glucose disposal rates (GDR)), exercise capacity (VO_2max_, maximal oxygen consumption), abdominal adiposity (computed tomography scans), cholesterol, and triglycerides. Results showed that body composition (body weight, subcutaneous adiposity), insulin sensitivity, VO_2max_, and cholesterol all improved (*p* < 0.05). HYPO decreased the percentage change in TMAO compared to an increase after EU (HYPO: −31 ± 0.4% vs. EU: 32 ± 0.6%, *p* = 0.04). Absolute TMAO levels were not impacted (HYPO: *p* = 0.09 or EU: *p* = 0.53 group). The change in TMAO after intervention was inversely correlated with baseline visceral adipose tissue (r = −0.63, *p* = 0.009) and GDR (r = 0.58, *p* = 0.002). A hypocaloric diet and exercise approach appears to be effective in reducing TMAO. Larger trials are needed to support this observation.

## 1. Introduction

Trimethylamine N-oxide (TMAO) was identified through untargeted metabolomic studies as a small molecule present in human systemic circulation that promotes cardiovascular disease [1]. More recently, TMAO has been implicated in the pathogenesis of obesity [2], insulin resistance [3,4,5,6], and renal disease [7,8]. Prospective clinical studies have shown that TMAO is predictive of adverse cardiovascular events, including myocardial infarction, stroke, and death [9]. In addition, several recent meta-analyses of clinical studies for TMAO all confirm circulating levels predict both cardiovascular disease and mortality risks, with each 10 µM increase in TMAO level being associated with an approximate 7.6% increase in relative risk of all-cause mortality [10,11,12]. A wealth of both animal model and clinical observation studies suggest that the gut microbiota-dependent TMAO pathway is an important participant in the development of cardiovascular and metabolic diseases [1].

The biosynthesis of TMAO is unique from traditional cardiovascular disease risk factors, in that its production is dependent on gut microbial metabolism. The primary precursors of TMAO are derived from dietary sources of phosphatidylcholine, choline, and L-carnitine. Once in the digestive tract, gut microbes aid in the catabolism of these compounds resulting in the production of trimethylamine (TMA) as a metabolic by-product. TMA is rapidly absorbed into the portal circulation and oxidized by hepatic enzymes, most notably flavin monooxygenase 3 (FMO3), which results in TMAO production [1,13]. TMAO re-enters the systemic circulation and contributes to the activation of endogenous macrophage foam cells that participate in the development of atherosclerosis [1,11,14,15].

There is a growing interest in identifying novel therapeutic approaches that reduce TMAO levels and/or block its production, particularly in obese individuals [2,16] given that adipose tissue expresses FMO3, the host enzyme responsible for synthesizing TMAO from gut microbiota [2]. While drug development studies targeting the gut microbial TMAO generating pathway have recently shown promise in attenuating atherosclerosis [17], and both platelet hyper-responsiveness and in vivo thrombosis potential in animal models [18], the most effective treatment modality remains unknown [19]. Brief oral courses of poorly absorbed antibiotics have also been shown to suppress TMAO generation in both mice and humans [1,9,20,21], but chronic antibiotic exposure results in antibiotic resistant microbial strains colonizing the intestines (and rebound in TMAO levels to pre-antibiotic levels) [1] and potentially explain the limited success of chronic antibiotics as a cardiovascular disease treatment [22,23,24]. Lifestyle modification including exercise and/or diet, on the other hand, is a leading treatment candidate for impacting TMAO concentrations given the profound effects on cardio-metabolic health. Indeed, chronic exercise reduces cardiovascular disease risk [25], as well as slows the progression of type 2 diabetes [26] in adults with prediabetes. Interestingly, TMAO levels are increased in diabetics compared to non-diabetics [2,27]. To date, the effects of supervised exercise training, with or without dietary caloric restriction, on circulating TMAO levels in obese humans has not yet been reported. Therefore, this study objective was to assess the impact of a 12-week lifestyle intervention on circulating TMAO levels in obese adults. Given that a caloric deficit improves obesity-related cardiometabolic risk factors [28], we sought to compare two different lifestyle approaches differing in energy balance: (1) exercise combined with a hypocaloric diet, and (2) exercise combined with a eucaloric diet. It was hypothesized that exercise combined with a hypocaloric diet would be more effective for reducing TMAO levels than exercise combined with a eucaloric diet. Relationships between TMAO changes and additional disease risk factors were examined, including body fat, glucose metabolism, and cardiovascular disease (CVD) risk factors.

## 2. Materials and Methods

### 2.1. Study Design

This was a post-hoc analysis of samples collected from previously conducted randomized-controlled trials investigating the effects of lifestyle interventions on cardiometabolic risk factors [28,29]. We had pre- and post-intervention plasma samples for sixteen adults with obesity (age: 66.1 ± 4.4 years; BMI (body mass index): 35.9 ± 5.3 kg/m^2^). The participants had been randomly assigned to either a 12-week eucaloric (EU) diet combined with exercise training, or a hypocaloric (HYPO) diet combined with exercise training. Anthropometric and metabolic data were collected before and after either the eucaloric or hypocaloric intervention. During testing phases, participants reported to the Clinical Research Unit for a 3-day metabolic control period. Activity and diet were closely monitored during this period. As previously described, a weight-maintenance diet with the following macronutrient composition (55% carbohydrate, 35% fat, and 10% protein) was provided to participants. Daily caloric intake was calculated as resting metabolic rate × 1.3 [29]. Metabolic testing procedures were performed after an approximate 12-h overnight fast.

### 2.2. Participants

Participants were weight stable (<2 kg weight change within the past 6 months), sedentary (<20 min of exercise twice per week), and free of medications known to affect study outcomes. Prior to study enrollment, participants underwent a medical history, physical examination, oral glucose tolerance test (OGTT), safety labs, and 12-lead echocardiogram. Participants were recruited from the local community and came from larger previously published cohorts [28,29]. Previous study protocols were approved by our Institutional Review Board and all participants provided written informed consent prior to study participation.

### 2.3. Lifestyle Intervention

Exercise: The exercise program was equivalent between the eucaloric and hypocaloric groups. Prior to initiating the exercise program, maximal oxygen consumption (VO_2max_) and maximal heart rate (HR_max_) were measured using an incremental, graded treadmill exercise test. To be considered a maximal exercise test, participants were required to achieve three of the following four criteria: oxygen consumption plateau (<150 mL/min), heart rate within 15 beats of age-predicted HR_max_, respiratory exchange ratio >1.15, and/or volitional fatigue. Exercise training occurred at a frequency of 5 days/week, for 50–60 min in duration, on either a treadmill or cycle ergometer. Initial exercise sessions were performed at an intensity between 60–65% HR_max_. Intensity was gradually increased across sessions so that by week 4, participants were maintaining an intensity of 80–85% HR_max_. VO_2max_ and HR_max_ were re-tested in 4-week increments (baseline, 4, 8, and 12 weeks) in order to monitor fitness improvements and adjust training intensity. All exercise sessions were supervised by an exercise physiologist. Polar heart rate monitors were used during all exercise sessions to confirm compliance to training intensity. The last exercise bout was completed approximately 16 h prior to post-testing assessments.

Diet: Participants in the eucaloric group were counseled not to alter their energy intake, while participants in the hypocaloric group were counseled to reduce daily calories by 500 kcal. In order to facilitate the caloric deficit, participants met with a nutritionist on a weekly basis. Energy expenditure was estimated by the Harris–Benedict equation, using an activity factor of 1.3 [30]. Dietary adherence was monitored via 3-day food recalls, which were completed prior to the intervention, as well as at weeks 1, 3, 6, 9, and 12. Bodyweight was monitored weekly to confirm compliance.

### 2.4. Body Composition

Height and weight measurements were taken after approximately 12 h of fasting, and participants were required to wear a standard hospital gown. Height was measured using a stadiometer and bodyweight was measured using a calibrated scale. An average of three measurements for both height and weight are reported. BMI was calculated from these data. Abdominal adiposity was measured with computerized axial tomography (CT) scans (Picker PQ6000 Scanner; Marconi/Picker Highland Heights, OH, USA). Cross-sectional images were taken at the L4 region of the abdomen. Regions of interest included subcutaneous and visceral depots.

### 2.5. TMAO Measurement

Fasting blood samples were collected in EDTA tubes, processed to isolate plasma, and frozen at −80 °C until analysis. TMAO levels were measured using stable isotope dilution liquid chromatography with online tandem mass spectrometry using an 8050 triple quadrupole mass spectrometer (Shimadzu Scientific Instruments, Columbia, MD, USA) as previously described [1,31]. The TMAO assay showed good inter- and intraday stability (CVs < 7%), accuracy (>98.5%), and stability across freeze–thaw cycles (intercycle CV < 9%) [31].

### 2.6. Glucose Metabolism

Oral glucose tolerance test (OGTT): Participants consumed a standard drink (75 g glucose). Blood samples were collected at the following time points: 0, 30, 60, 90, 120, and 180 min. Samples were analyzed for glucose and insulin. Total area-under-the-curve (AUC) was a calculated using the trapezoidal method. Plasma glucose was analyzed using YSI 2300 STAT Plus analyzer (Yellow Springs, OH, USA). Plasma insulin was analyzed by radioimmunoassay (Millipore, Billerica, MA, USA).

Euglycemic-hyperinsulinemic clamp: Plasma glucose was clamped at 90 mg/dL during hyperinsulinemia (40 mU/m^2^/min^−1^), as previously described [32,33]. A primed-continuous insulin infusion was carried out over 120 min via catheter placed in the antecubital vein. Glucose (dextrose 20%) was concurrently infused, while the contralateral hand was warmed to 60 °C for the purpose of arterializing blood. Plasma glucose was assessed every five minutes, and this value was used to determine subsequent glucose infusion rates. Glucose disposal rate (GDR) during the 90–120 min time increment of the clamp were reported to determine insulin sensitivity.

### 2.7. Statistical Analysis 

Data are presented as mean ± standard deviation, unless otherwise noted. Data were analyzed using GraphPad Prism (v7, San Diego, CA, USA). Baseline characteristics between groups were analyzed using Student’s unpaired *t* test. Within group changes were analyzed by Student’s paired *t* test to test treatment effects. Comparison of between group differences (HYPO: *n* = 7 vs. EU: *n* = 9) was accomplished by repeated measures ANOVA. Secondarily, we compared percent change between interventions using Student’s unpaired *t* test. Due to missing data, two post-test data points were imputed using a group average approach (HYPO: *n* = 1; EU: *n* = 1). Relationships between variables were determined using Pearson’s correlation coefficient. Hierarchical linear regression was used to statistically control for baseline TMAO levels after statistical significance was detected between percent change in TMAO and metabolic outcomes (SPSS, IBM v25, Armonk, NY, USA). Data were tested for normality using Shapiro–Wilk test, and were log transformed when non-normally distributed for correlation analyses. Significance was accepted at *p* < 0.05.

## 3. Results

### 3.1. Lifestyle Intervention

Participant characteristics are shown in Table 1. There were no baseline differences for any baseline between eucaloric and hypocaloric groups. The lifestyle intervention significantly improved VO_2max_ in both eucaloric (pre: 19.7 ± 2.5 vs. post: 22.4 ± 3.1 mL/kg/min; *p* = 0.005) and hypocaloric (pre: 21.9 ± 2 vs. post: 25.2 ± 2.0 mL/kg/min; *p* = 0.013) groups. As expected, the magnitude of improvement was not different between groups (EU: 14.1 ± 11.7% vs. HYPO: 15.5 ± 11.7%, between group: *p* = 0.81).

Average kcal/day were reduced by ~500 kcal in the hypocaloric group (pre: 1976.6 ± 273.4 vs. post: 1403.9 ± 190.1 kcal; *p* = 0.001), while the eucaloric group remained at maintenance calories (pre: 1956 ± 818.9 vs. post: 1864.8 ± 855.7; *p* = 0.254). Body weight and BMI were significantly reduced in both groups, as shown in Table 1. While not statistically different, the percent reduction in body weight tended to be larger in the hypocaloric versus eucaloric group (HYPO: −7.8 ± 1.1% vs. EU: −5.7 ± 2.6%; *p* = 0.06).

### 3.2. TMAO Concentrations

**Figure 1:**
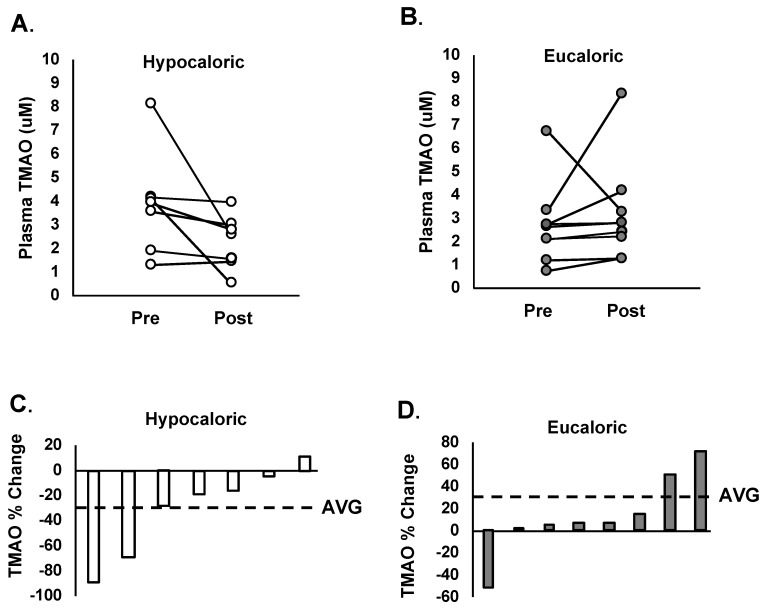
(**A**) Individual changes in plasma TMAO (trimethylamine N-oxide) concentrations before and after 12 weeks of a hypocaloric diet combined with supervised exercise. (**B**) Individual changes in plasma TMAO concentrations before and after 12 weeks of a eucaloric diet combined with supervised exercise. (**C**) Individual percent change in plasma TMAO concentrations after 12 weeks of a hypocaloric diet combined with supervised exercise. Group average denoted by a horizontal dashed line. (**D**) Individual percent change in plasma TMAO concentrations after 12 weeks of a eucaloric diet combined with supervised exercise. Group average denoted by a horizontal dashed line.

Baseline TMAO levels were not different between hypocaloric or eucaloric groups (HYPO: 3.2 ± 1.9 vs. EU: 2.7 ± 1.7 µM; *p* = 0.260). In response to the interventions, absolute levels of TMAO did not significantly change within the hypocaloric (pre: 3.8 ± 2.2 vs. post: 2.2 ± 1.3 µM; *p* = 0.136) or eucaloric (pre: 2.7 ± 1.7 vs. post: 3.2 ± 2.3 µM; *p* = 0.537) groups. However, Figure 1 displays individual changes in plasma TMAO concentrations after the hypocaloric (Figure 1A), and eucaloric (Figure 1B) interventions, and shows considerable variation. While the change in absolute levels of TMAO were not significantly different between groups (F = 2.421, *p* = 0.132), the average percent change in TMAO was significantly different between groups (EU: 32 ± 0.6% vs. HYPO: −31 ± 0.4%, *p* = 0.04, Figure 1C,D).

### 3.3. Glucose Metabolism

Oral glucose tolerance test: Fasting glucose was significantly reduced in the hypocaloric group, while the eucaloric group remained unchanged, as shown in Table 1. Glucose-AUC_180min_ responses trended towards a reduction in the hypocaloric group (pre: 24,555 ± 2549 vs. post: 21,608 ± 2352 mg/dL × 180 min; *p* = 0.071) and remained unchanged in the eucaloric group (pre: 25,821 ± 6127 vs. post: 24,064 ± 3696 mg/dL × 180 min; *p* = 0.173). Fasting insulin trended toward a reduction in both groups, as shown in Table 1. Insulin AUC_180min_ responses tended to decrease in both the hypocaloric (pre: 10,432 ± 4577 vs. post: 6,469 ± 1870 µU/mL × 180 min; *p* = 0.079) and eucaloric (pre: 15,162 ± 9912 vs. post: 11,520 ± 7236 µU/mL × 180 min; *p* = 0.075) groups, although this effect was not statistically significant.

**Figure 2:**
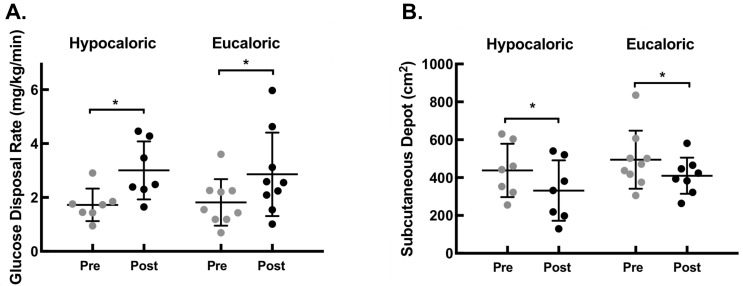
(**A**) Individual glucose disposal rates during 90–120 min time increment of euglycemic-hyperinsulinemic clamp before and after the 12-wk lifestyle intervention, which increased in both hypocaloric and eucaloric groups. Group average denoted by horizontal line, with standard deviation bars. (**B**) Individual computed tomography-measured subcutaneous fat depots before and after 12-wk lifestyle intervention, which decreased in both hypocaloric and eucaloric groups. Group average denoted by horizontal line, with standard deviation bars. * indicates *p* < 0.05.

Euglycemic-hyperinsulinemic clamp: Peripheral insulin sensitivity, as assessed by glucose disposal rates, significantly improved in both the hypocaloric and eucaloric groups, as shown in Figure 2A. The magnitude of improvement was not different between groups (HYPO: 83.6% vs. EU: 59.6%; *p* = 0.41).

### 3.4. Abdominal Adiposity

Subcutaneous abdominal fat depot significantly decreased in both the hypocaloric and eucaloric groups, as shown in Figure 2B. The hypocaloric group experienced a larger percent reduction than the eucaloric group (HYPO: −27% vs. EU: −10%; *p* = 0.013).

The visceral abdominal fat depot was significantly decreased in the eucaloric group (pre: 174.9 ± 118 vs. post: 138.9 ± 98 cm^2^; *p* = 0.025), while the hypocaloric group was unchanged (pre: 255.7 ± 96 vs. post: 232 ± 67 cm^2^; *p* = 0.434). The relative decrease was not different between groups (HYPO: −6.1% vs. −16.3%; *p* = 0.156).

### 3.5. Lipid Profile

Cholesterol was significantly reduced in the hypocaloric group, while the eucaloric group trended towards a significant reduction, as shown in Table 1. The relative decrease was not different between groups (HYPO: 9.2% vs. EU: 7.5%; *p* = 0.752). In addition, triglycerides were significantly reduced in the eucaloric but not hypocaloric group, as shown in Table 1. The relative decrease was not different between groups (HYPO: −5.7% vs. EU: −19.6%; *p* = 0.233).

### 3.6. Correlation Analysis

**Figure 3:**
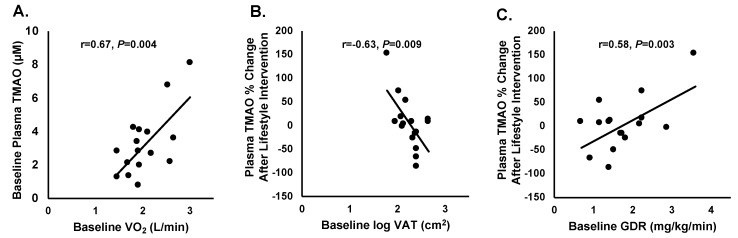
(**A**) Correlation between baseline plasma TMAO (µM) and baseline VO_2max_ (L/min) (r = 0.67, *p* = 0.004). (**B**) Correlation between baseline log transformed visceral adipose tissue (VAT) and percent change in TMAO after the lifestyle intervention (r = −0.63, *p* = 0.009). (**C**) Correlation between baseline glucose disposal rate and percent change in TMAO after the lifestyle intervention (GDR (glucose disposal rate): r = 0.58, *p* = 0.003).

Baseline absolute VO_2max_ was significantly correlated with baseline levels of TMAO, as shown in Figure 3A, (r = 0.67, *p* = 0.004). Change in TMAO after lifestyle intervention was inversely correlated with baseline visceral adipose tissue (r = −0.63, *p* = 0.0094), and after statistically controlling for baseline TMAO levels, was r = −0.54 (*F change* = 8.433, *p* = 0.012). No other baseline factors were related with baseline TMAO (Table S1). In addition, change in TMAO after lifestyle intervention correlated with baseline GDR (90–120 min mg/kg/min) (r = 0.58, *p* = 0.002), and after statistically controlling for baseline TMAO levels was r = 0.52 (*F change* = 7.483, *p* > 0.05). No correlations were observed with the change in TMAO and the change in any body composition or metabolic outcome (Table S2).

## 4. Discussion

The primary finding of this study is that caloric restriction combined with exercise promoted a reduction in TMAO, while a eucaloric diet combined with exercise promoted a modest increase, in this modest sized cohort of older obese, insulin-resistant adults. Previous studies examining the effects of lifestyle interventions on TMAO are scarce. One study reported that three months of dietary modification combined with recommendations to exercise had no effect on TMAO levels in 34 obese adults preparing for bariatric surgery [34]. Similarly, a second study reported no change in TMAO levels in 220 adults after nine months of dietary counseling combined with recommendations to exercise [35]. However, interestingly, in that study, subjects who experienced the greatest reduction in carotid artery intimal medial thickness were those demonstrating the largest reduction in TMAO levels [35]. It is difficult to interpret the effects of exercise on TMAO from these studies, as subjects were only given recommendations to increase physical activity levels. The present study is the first to document the effects of a highly-controlled supervised exercise intervention. An advantage of our approach is that we were able to accurately determine compliance, including frequency (days per week), duration of each exercise session, and training intensity (confirmed through heart rate monitoring). Our findings extend previous work, and when expressed as percent change after intervention, these data suggest that dietary-induced caloric restriction combined with exercise may be more robust for altering TMAO, versus an eucaloric diet plus exercise.

These findings are in alignment with the TMAO production pathway. It can be posited that an indirect benefit of the hypocaloric diet used in this study led to a reduction in absolute levels of dietary precursors, partially explaining the observed changes in TMAO. Circulating levels of TMAO are sensitive to change through manipulation of dietary precursors, as this approach has been used previously as an experimental maneuver described as a phosphatidylcholine challenge [9]. Results from this challenge show increased plasma TMAO levels after ingestion of two large hard-boiled eggs combined with deuterium-labeled phosphatidylcholine [9]. Herein, we consistently show a potential link between dietary contributions to TMAO levels in humans. Whether TMAO changes were driven by a negative energy balance, or by absolute reductions in dietary precursors associated with adhering to a caloric restriction protocol is unknown, and larger trials with rigorous dietary control will be needed to address these issues.

TMAO production is dependent on gut microbial metabolism creating novel opportunities to therapeutically target the microbiome. It is well accepted that environmental exposures such as exercise and diet contribute to the composition of the gut microbiome. While exercise has been shown to alter gut microbiome distribution and diversity [36,37], the current study showed that 12 weeks of supervised exercise in the eucaloric diet arm did not significantly reduce TMAO levels. It is notable that prior studies have suggested that inter-individual variations in TMAO level responses to animal source foods depends upon the starting microbial composition [38]. The lack of effect of exercise on TMAO levels, might similarly be explained by inter-individual variation in gut microbial community composition [38]. Although baseline TMAO differences were not observed between groups (*p* = 0.26), we cannot rule out that a ‘bottom effect’ may be limiting the magnitude of decrease within the eucaloric arm. Systemic monitoring of TMAO levels during lifestyle intervention might permit a more personalized approach toward lifestyle intervention with improved reduction in cardiovascular and metabolic risks, though further studies with hard outcomes are needed in this area. Although exercise alone did not significantly reduce TMAO, it is important to note that numerous alternative beneficial effects occurred in the eucaloric arm of the study, including reductions in body weight, abdominal adiposity, and as well as improvements in peripheral insulin sensitivity, triglycerides, and cholesterol. Thus, exercise remains an effective treatment against cardio-metabolic diseases and future studies should continue to explore the impact of exercise on the gut microbiome in order to assess the significance of TMAO metabolism.

A noteworthy observation is that both intervention approaches led to comparable improvements in several health-related outcomes, while the direction of TMAO responses differed between the groups. Specifically, both hypocaloric and eucaloric groups experienced reductions in body weight and abdominal adiposity, as well as improvements in aerobic capacity and peripheral insulin sensitivity. Consistent with our findings, a study of 220 subjects undergoing lifestyle intervention showed improvements in several cardiovascular risk factors, while TMAO levels were unchanged [35]. In addition, a secondary analysis of the PREDIMED trial reported an inverse relationship between TMAO levels and type 2 diabetes risk after one year of dietary intervention in 694 subjects [39]. Interestingly, our correlation analysis is consistent with this later report in that we show people who have less visceral fat and who are more insulin sensitive have smaller reductions in TMAO post-treatment. However, we collectively see that on average our treatments reduce cardiovascular disease risk factors in both hypocaloric and eucaloric exercise interventions. Taken together, these findings suggest that improvements in cardio-metabolic health can be realized in the presence or absence of TMAO reductions based on the subject population. Given the role of TMAO in atherosclerotic development, it is possible that stronger relationships between TMAO after lifestyle intervention and vascular outcomes could be seen in patients with diagnosed cardiovascular or renal disease.

The current study is not without limitations that warrant mention. This was a post-hoc analysis in which we analyzed a sample of participants from previously conducted randomized controlled trials. We acknowledge that the sample size was modest. However, given the novelty of our primary outcome (human TMAO concentrations) it was not possible to complete a priori power calculations to guide sample size decisions. We observed significant differences between interventional approaches only when data were expressed as percent change. This could be partially attributed to large inter-individual variability in TMAO responses. Similarly, there is some evidence that factors such as sex and FMO3 genetic variants can influence TMAO levels [13,40]. It is notable that recent meta-analyses of clinical TMAO studies indicate that per 10 uM change (increase) in TMAO, there is a corresponding 7.6% increase in overall mortality risk [10]. Whether those that achieved TMAO reduction with the present interventions benefitted will require long-term outcome trials. Another limitation of the present study is precise measurements of known dietary precursors of TMAO were not performed, and such analyses could possibly have strengthened our conclusions. Given the study design, it is not possible to assess the temporality of TMAO changes, so the acute versus chronic effects of lifestyle-induced changes on TMAO remains unknown. Nonetheless, these novel data provide insight that can be used to inform the development of future studies testing the effects of lifestyle modification on TMAO in humans.

Through this post-hoc analysis, we found that diet-induced caloric restriction combined with supervised exercise may be more effective for reducing circulating TMAO, compared to exercise combined with a eucaloric diet. This may suggest that TMAO levels are more amenable to change through dietary-induced caloric restriction versus an exercise effect alone. However, we observed that health improvements occurred in the absence of a significant TMAO reduction, suggesting that exercise acts, in part, through the TMAO-independent pathway in obese, insulin-resistant adults. Randomized controlled trials with larger samples sizes will be needed to confirm our findings. Future studies should also aim to tease out the impact of negative energy balance versus reductions in absolute levels of TMAO in dietary precursors for promoting beneficial changes in TMAO levels and reducing cardio-metabolic disease risk.

## Figures and Tables

**Table 1:** Participant characteristics before and after lifestyle intervention.

	Hypocaloric (*n* = 7)			Eucaloric (*n* = 9)		
	Pre	Post	*p* Value	Pre	Post	*p* Value
Age (years)	67 ± 5	-	-	65 ± 4	-	-
Sex (M/F)	2/5	-	-	3/6	-	-
Body Weight (kg)	100 ± 16	91 ± 14	<0.05	100 ± 19	94 ± 17	<0.05
BMI (kg/m^2^)	35 ± 5	32 ± 5	<0.05	37 ± 5	35 ± 5	<0.05
FPG (mg/dL)	106 ± 8	100 ± 10	0.02	106 ± 20	103 ± 14	0.26
2-h PPG (mg/dL)	160 ± 22	146 ± 21	0.16	175 ± 45	154 ± 30	0.16
FPI (µU/mL)	17 ± 7	13 ± 5	0.05	19 ± 12	14 ± 6	0.06
2-h Insulin (µU/mL)	89 ± 52	71 ± 61	0.08	104 ± 60	87 ± 77	0.512
Triglycerides (mg/dL)	161 ± 70	146 ± 71	0.49	168 ± 51	134 ± 43	0.009
Cholesterol (mg/dL)	200 ± 19	181 ± 22	0.04	206 ± 24	191 ± 30	0.05

Data are displayed as mean ± standard deviation. BMI: body mass index; FPG: fasting plasma glucose; PPG: postprandial glucose; FPI: fasting plasma insulin; -: statistical test not applicable.

## Supplementary Materials

The following are available online at http://www.mdpi.com/2072-6643/11/1/179/s1, Table S1: Pearson correlation analyses, Table S2: Pearson correlation analyses.

## Clinical Trial Registration

This study was not registered as a clinical trial, this was a secondary analysis.
