# Supplementary material for: Effects of Lifestyle Intervention on Plasma Trimethylamine N-Oxide in Obese Adults
Source: Nutrients. 2019 Jan 16;11(1):179. doi: 10.3390/nu11010179 (PMC6356515; doi:10.3390/nu11010179)
Supplement: Supplementary file 1 [file nutrients-11-00179-s001.pdf]

Supplementary Material

## Supplementary Table S1: Pearson correlation analyses.

| Baseline Plasma TMAO (μM) vs: | r | *p* |
|---|---|---|
| BMI (kg/m$^2$) | 0.191 | 0.479 |
| Age (yrs) | 0.413 | 0.118 |
| VAT (cm$^2$) | 0.189 | 0.484 |
| GDR (mg/kg/min) | -0.130 | 0.632 |
| Glucose $AUC_{180min}$ (mg/dLx180 min) | -0.071 | 0.792 |
| Insulin $AUC_{180min}$ (μU/dLx180 min) | 0.096 | 0.725 |

BMI: body mass index; VAT: visceral adipose tissue; GDR: glucose disposal rate; $GlucoseAUC_{180}$: 180 min glucose-area-under the curve from oral glucose tolerance test; $InsulinAUC_{180}$: 180 min insulin-area-under-the curve from oral glucose tolerance test. Non-normally distributed data were log transformed (VAT, $GlucoseAUC_{180}$ $InsulinAUC_{180}$).

## Supplementary Table S2: Pearson correlation analyses.

| Plasma TMAO % Change After Lifestyle Intervention vs: | r | *p* |
|---|---|---|
| BMI (kg/m$^2$) | 0.134 | 0.621 |
| Age (yrs) | -0.126 | 0.642 |
| Glucose $AUC_{180min}$ (mg/dLx180 min) | -0.012 | 0.964 |
| Insulin $AUC_{180min}$ (μU/dLx180 min) | -0.234 | 0.379 |
| $VO_2$ max (L/min) | -0.435 | 0.092 |

BMI: body mass index; $GlucoseAUC_{180}$: 180 min glucose-area-under the curve from oral glucose tolerance test; $InsulinAUC_{180}$: 180 min insulin-area-under-the curve from oral glucose tolerance test. Non-normally distributed data were log transformed (VAT, $GlucoseAUC_{180}$ $InsulinAUC_{180}$).
